# Efficacy of vaginal natural orifice transluminal endoscopic sentinel lymph node biopsy for endometrial cancer: a prospective multicenter cohort study

**DOI:** 10.1097/JS9.0000000000000551

**Published:** 2023-06-19

**Authors:** Li Deng, Yi Liu, Yuanyan Yao, Yuan Deng, Shuai Tang, Li Sun, Yanzhou Wang

**Affiliations:** a Department of Obstetrics and Gynecology, The First Affiliated Hospital (Southwest Hospital), Army Medical University, Chongqing; b Departments of Gynecological Oncology, National Cancer Center/National Clinical Research Center for Cancer/Cancer Hospital l & Shenzhen Hospital, Chinese Academy of Medical Sciences and Peking Union Medical College, Shenzhen; c Departments of Gynecological Oncology, National Cancer Center/National Clinical Research Center for Cancer/Cancer Hospital, Chinese Academy of Medical Sciences and Peking Union Medical College, Beijing, China

**Keywords:** comprehensive surgical staging, endometrial cancer, sentinel lymph nodes, vNOTES

## Abstract

**Introduction:**

Natural orifice transluminal endoscopic surgery (NOTES) is performed increasingly often despite the lack of high-quality evidence confirming its safety, especially for malignant diseases. The aim of this prospective study is to verify that vaginal NOTES (vNOTES) can be performed safely and effectively in early endometrial cancer staging surgery.

**Materials and Methods:**

This prospective study was conducted in two tertiary hospitals in southern China from January 2021 to May 2022. A total of 120 patients with stage I endometrial cancer were included. vNOTES or multiport laparoscopic staging surgery was selected according to each patient’s wishes. The primary outcome was the sentinel lymph node detection rate, analyzed by a noninferiority test. The secondary outcomes were perioperative outcomes.

**Results:**

Among the 120 patients enrolled, 57 underwent vNOTES, and 63 underwent multiport laparoscopy. The patient-specific detection rates of sentinel lymph nodes were 94.73 and 96.82% in the vNOTES and laparoscopy groups, respectively. Additionally, the bilateral detection rates were 82.46 and 84.13%, and the side-specific detection rates were 88.60 and 90.48% in these two groups, respectively. All three detection rates in the vNOTES group were noninferior to those of laparoscopy group at a noninferiority cutoff of –15%. The median operation times of the vNOTES and laparoscopy groups were 132.35 and 138.73 min (*P*=0.362), and the median estimated blood loss were 75 and 50 ml (*P*=0.096), respectively. No intraoperative complications occurred in either group. The pain scores on the Numerical Rating Scale at both 12 h and 24 h after operation were significantly lower in the vNOTES group (*P*<0.001) and the median postoperative hospital stay was significantly shorter in the vNOTES group (*P*=0.001).

**Conclusion:**

This study illustrates the potential applicability of vNOTES in gynecological malignancy surgery by demonstrating its safety and effectiveness in endometrial cancer staging. However, its long-term survival outcomes require further exploration.

## Introduction

Highlights

- Pelvic and abdominal sentinel lymph nodes can be detected accurately by vaginal NOTES (vNOTES).
- vNOTES gets similar perioperative outcomes with conventional laparoscopy.
- vNOTES for malignancy represents an expanded scope of the developed technology.

Staging surgery is an important strategy for the treatment of early endometrial cancer. The National Comprehensive Cancer Network and European Society of Radiology and Oncology both recommend minimally invasive surgery as the preferred surgical approach for early endometrial cancer staging, including multiport laparoscopy, single-site laparoscopy, and robot-assisted laparoscopy, which have been studied extensively^1–3^. Gynecologists have also used the transvaginal route to perform endometrial cancer staging; this approach minimized trauma but lacked the ability to assess lymph nodes^4^.

Natural orifice transluminal endoscopic surgery (NOTES) techniques have been further improved in recent years and increasingly performed in urological, general, and gynecological surgeries. In vaginal NOTES (vNOTES), the advantages of vaginal surgery and laparoscopic surgery are combined, allowing for the evaluation of lymph nodes, and making it possible to apply NOTES technology for malignant tumors. The application of vNOTES technology in endometrial cancer was first reported in a case report in 2014^5^. Since then, similar operations have been performed at several medical institutions. There have been some very innovative case reports and videos^6–8^, as well as some studies with a small sample size^9,10^. Huber and Hurni^9^ conducted a preliminary study of vNOTES sentinel lymph node biopsy (SLNB) in five endometrial cancer patients, and Lee *et al.* ^10^ conducted a retrospective observational study of 15 endometrial cancer patients with sentinel lymph node mapping or pelvic lymph node dissection; both studies confirmed the feasibility and safety of this surgical procedure.

We have been using vNOTES to treat endometrial cancer since August 2017 at our institute. Among the initially reported 15 cases, 12 patients accepted pelvic lymph node ± paraaortic lymph node dissection, and 3 patients accepted sentinel lymph node mapping^11^. Through these were preliminary explorations, we realized that SLNB can better balance the benefits and risks of vNOTES and reflect the minimally invasive concept to the utmost extent. To further explore the clinical value of vNOTES in endometrial carcinoma, we conducted a retrospective cohort study of 74 cases in 2021 and found that vNOTES endometrial cancer staging with SLNB appeared comparable to conventional laparoscopy in terms of safety and efficacy; however, vNOTES reduced visible scarring, shortened hospital stays, and led to fewer perioperative complications^12^. Nevertheless, these studies were retrospective and lacked high-level evidence to further confirm that this surgical approach can be used more widely worldwide. Therefore, we conducted a prospective cohort study in two tertiary hospital tumor centers to compare the clinical outcomes of vNOTES endometrial cancer staging with SLNB between vNOTES and conventional laparoscopy.

## Materials and methods

This study was a prospective open-label cohort trial comparing vNOTES and traditional laparoscopy applied in the staging surgery of endometrial cancer in two tertiary hospitals in the southern region of China. The study was conducted in accordance with applicable regulatory requirements and the principles of the Declaration of Helsinki. Approval was obtained from the ethics committee/Institutional Review Board of the First Affiliated Hospital of Army Medical University (KY2020094) and Shenzhen Hospital, Chinese Academy of Medical Sciences, and Peking Union Medical College (KYKT2021-9-2). The operations were performed by two surgeons (WYZ and SL) who are both experts in gynecologic surgical treatment and equally skilled in operating with both techniques. The study was registered in the Chinese Clinical Trial Registry (registration number: ChiCTR2000040546 https://www.chictr.org.cn/showproj.aspx?proj=57936). The work has been reported in line with the STROCSS criteria^13^.

### Patients

The patients were selected according to the following inclusion criteria: at least 18 years of age; histologically confirmed endometrial adenocarcinoma on preoperative endometrial biopsy; confirmation of stage I disease by MRI; no tumor mass larger than 2 cm in the greatest dimension; patient accepted minimally invasive staging surgery; WBC greater than 3.0×10^9^/l; platelets greater than 100×10^9^/L; creatinine less than 2.0 mg/dl; bilirubin less than 1.5 times the normal upper limit; AST/SGOT or ALT/SGPT less than three times the normal upper limit; ECOG less than or equal to 1; and no other malignant tumor in the last 5 years. The exclusion criteria were as follows: previous pelvic or abdominal radiotherapy; the largest diameter of the uterus was greater than 12 cm; contraindications to surgery; and inadequate follow-up visits because of poor compliance or significant travel requirements. The recruitment of patients began in January 2021 and ended in May 2022.

### Selection of surgical procedure

The vNOTES and multiport laparoscopy surgical procedures, as well as their advantages and disadvantages, were carefully described by researchers to patients. The patients decided which surgical method to undergo and subsequently signed the surgical consent form.

### Sentinel lymph node mapping

#### Carbon nanoparticles (CNPs) as lymphatic tracers

A CNP suspension (50 mg in 1 ml, Chongqing LUMMY Pharmaceutical Co.) was diluted to 4 ml (12.5 mg/ml) with sterile water and then injected into the cervix (1 ml submucosally and 1 ml at 1–2 cm deep) at the 3 o’clock and 9 o’clock positions. The injection at each position was completed in ~2 min and required local pressure to avoid extravasation. The operation began 15–20 min after the lymphatic tracer injection to allow better diffusion of the CNPs.

#### Indocyanine green as a lymphatic tracer

One 25 mg vial of indocyanine green (Dandong Yichuang Pharmaceutical Co., Ltd) was reconstituted in 10 ml of sterile water (2.5 mg/ml) and then injected into the cervix (0.5 ml submucosally and 0.5 ml at 1–2 cm deep) at the 3 and 9 o’clock positions. The operation was subsequently initiated without a waiting period with a fluorescence imaging system (Pinpoint Endoscopic Fluorescence Imaging System).

### vNOTES procedure

A total hysterectomy and bilateral adnexectomy were performed using vNOTES procedures in the vNOTES group, according to the descriptions in our previous article^12^. Here, we briefly describe the steps. After sealing the cervix by suturing, the anterior and posterior vaginal fornix were incised, and the pelvic cavity was accessed from the Douglas cul-de-sac to obtain peritoneal lavage fluid. The uterosacral and a portion of the cardinal ligaments were dissected to establish the surgical pass and pneumoperitoneum; then, total hysterectomy and bilateral adnexectomy were performed using vNOTES.

The peritoneum of the lateral pelvic wall was opened along the obturator umbilical artery to expose the lymph node drainage area. SLN mapping was started near the uterus, and SLNs were defined as the first group of developed lymph nodes along the stained lymphatic vessels. If no lymph nodes were identified, traditional laparoscopic surgery was immediately performed to identify and harvest the SLNs. If a side could not be mapped, systemic lymphadenectomy was performed laparoscopically. Routine pelvic drainage was not necessary.

### Laparoscopic surgery

Intraperitoneal exploration was performed after the trocars were placed, and the peritoneal lavage fluid was collected. The peritoneum of the lateral pelvic wall was opened along the iliac artery to expose the lymph node drainage area. SLN mapping, in the same way, was started near the uterus, and the SLNs were defined as the first group of developed lymph nodes along the stained lymphatic vessels. If a side could not be mapped, lymph node dissection was subsequently performed. Routine pelvic drainage was not necessary.

### Histopathologic procedures

According to the SLN mapping algorithm previously published by the Memorial Sloan-Kettering Cancer Center^14^, a single section of each removed lymph node was stained with hematoxylin and eosin (H&E) and evaluated for metastases. If a metastatic tumor was present, no further evaluation was performed. If the SLN was negative, ultrastaging was performed. Ultrastaging consisted of a corresponding cytokeratin (AE1:AE3) immunohistochemical stain of the original H&E sample; then, H&E and adjacent cytokeratin immunohistochemical staining were performed on additional sections at 50 microns into the block to detect possible metastases missed by the initial evaluation.

### Follow-up

Postoperative follow-up visits were conducted in the outpatient clinic at 1, 3, and 6 months after surgery. The follow-up program consisted mainly of gynecological examination and gynecological ultrasound; additional items could be added according to the demands of the situation.

### Outcomes

The primary endpoint was the patient-specific detection rate (PSDR) of the SLN, defined as the proportion of patients in which hemipelves SLN was identified by mapping. This outcome was selected because it was relevant to the impact of the different surgical pathways in stained SLN detection. Meanwhile, the side-specific detection rate (defined as the proportion of hemipelves in which an SLN mapped, SSDR), bilateral detection rate (defined as the proportion of patients in whom SLN mapped bilaterally, BDR), SLN locations, and histopathologic diagnosis for the specimen were also displayed to further illustrate the primary endpoint. The secondary endpoints were the perioperative outcomes, including the operation time, estimated blood loss, intraoperative and postoperative complications, postoperative pain scores, and hospitalization costs.

### Statistical analysis

**Figure 1:**
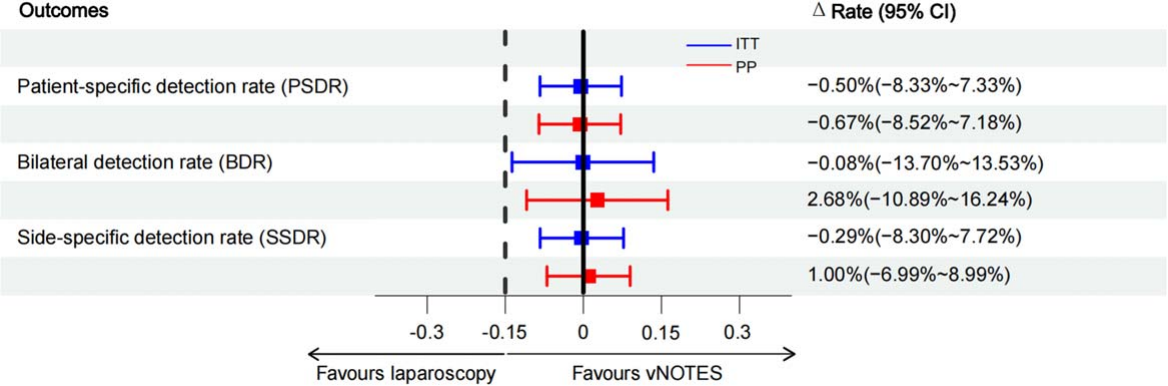
Subgroup analysis of the detection rate of sentinel lymph nodes by noninferiority comparison for different surgical approaches.

Based on the 92.2–95.7% detection rate via SLN mapping reported in our previous studies, we estimated that at least 84 subjects should be enrolled based on an expected detection rate of 95% in the PP (per-protocol) analysis using a 1:1 noninferiority test, with a CI of 95% and power level of 80%. Statistical analyses and the noninferiority assessment were based on the difference in detection rates between the vNOTES group and the multiport laparoscopy group. The patients who accepted traditional laparoscopic detection secondary to failed SLN detection using vNOTES were still included in the vNOTES group according to the protocol. The patients were switched to the laparoscopy group only when the vNOTES procedure could not be completed for other reasons, such as a large uterus or heavy adhesions. A sensitivity analysis was performed to exclude the compactness of the PP patients from the main outcome. Two-sided 95% CIs for differences were computed using the unstratified method to determine the normal approximation interval. For primary efficacy endpoints, the noninferiority of vNOTES to multiport laparoscopy was considered to be demonstrated if the lower limit of the two-sided 95% CI for the difference in detection rate was greater than –15%. Prespecified subgroup analyses evaluated the impact of baseline patient and disease characteristics, including BMI, uterus size, and previous abdominal surgery (Fig. 1).

For the other variables, we used multiple statistical methods to test for differences, including the χ^2^-test and Fisher’s exact test, which are reported as numbers and proportions. Student’s *t*-test was used for continuous variables with a normal distribution, which are presented as means ± SD, and the Mann–;Whitney *U* rank sum test was used for noncontinuous variables, which are presented as medians and interquartile ranges. Differences between the groups were considered statistically significant for values of *P*<0.05. The data were analyzed with SPSS 22.0 (IBM Corp.), R 4.0.3, and RStudio 1.1.383.

## Results

A total of 120 patients with endometrial cancer were enrolled in this prospective study; 57 patients were included in the vNOTES group and 63 patients were included in the laparoscopic group, and one patient converted from the vNOTES group to the laparoscopic group due to difficulty in hysterectomy caused by a large uterus. All of the patients underwent total hysterectomy with bilateral salpingo-oophorectomy and SLN mapping at the time of surgical staging. The patient characteristics, including median age, BMI, uterus size, grade, depth of myoinvasion, and stage, are detailed in Table 1. All patients had clinical stage I disease at their initial diagnosis, with final pathology demonstrating disease staging as follows: stage IA, 46 (80.70%); stage IB, 9 (15.79%); stage IIIA, 1 (1.75%) and stage IIIC1, 1 (1.75%) in the vNOTES group and stage IA, 57 (90.48%); stage IB, 4 (6.35%); stage IIIA, 2 (3.17%) in the laparoscopic group.

**Table 1:** Baseline patient characteristics and treatment-related variables.

Variable	vNOTES (*n*=57)	Multiport laparoscopy (*n*=63)	*P*
Age, years	51.46±7.83	52.52±8.47	0.476
<50 years, *n* (%)	23 (40.35)	22 (34.92)	0.539
≥50 years, *n* (%)	34 (59.65)	41 (65.08)	
BMI	26.25±3.09	25.76±4.10	0.462
≤24, *n* (%)	12 (21.05)	26 (41.27)	0.017
>24, *n* (%)	45 (78.95)	37 (58.73)	
Gravidity	3 (2–4)	3 (2–4)	0.535
Parity	2 (1–2)	1 (1–3)	0.164
Previous abdominal surgery, *n* (%)	18 (31.58)	25 (39.68)	0.355
Pelvic adhesions, *n* (%)	6 (10.53)	11 (17.46)	0.277
Maximum uterine width, cm	5.0 (4.35–5.85)	5.2 (4.4–6.5)	0.320
Histologic grade, *n* (%)			0.798
G1	34 (59.65)	34 (53.97)	
G2	21 (36.84)	27 (42.86	
G3	2 (3.51)	2 (3.17	
Tumor size, cm, *n* (%)			0.115
≤2	29 (50.88)	41 (65.08	
>2	28 (49.12)	22 (34.92	
SLN number	4 (2-5)	4 (2-7)	0.497
LVSI (+), *n* (%)	2 (3.51)	5 (7.94)	0.301
peritoneal lavage fluid (+), *n* (%)	2 (3.51)	4 (6.35)	0.476
Myometrial invasion, *n* (%)			0.107
None	4 (7.02)	10 (15.87)	
≤1/2	44 (77.19)	49 (77.78)	
>1/2	9 (15.79)	4 (6.35)	
FIGO stage, *n* (%)			0.247
IA	46 (80.70)	57 (90.48)	
IB	9 (15.79)	4 (6.35	
IIIA	1 (1.75)	2 (3.17)	
IIIC1	1 (1.75)	0	

**Figure 2:**
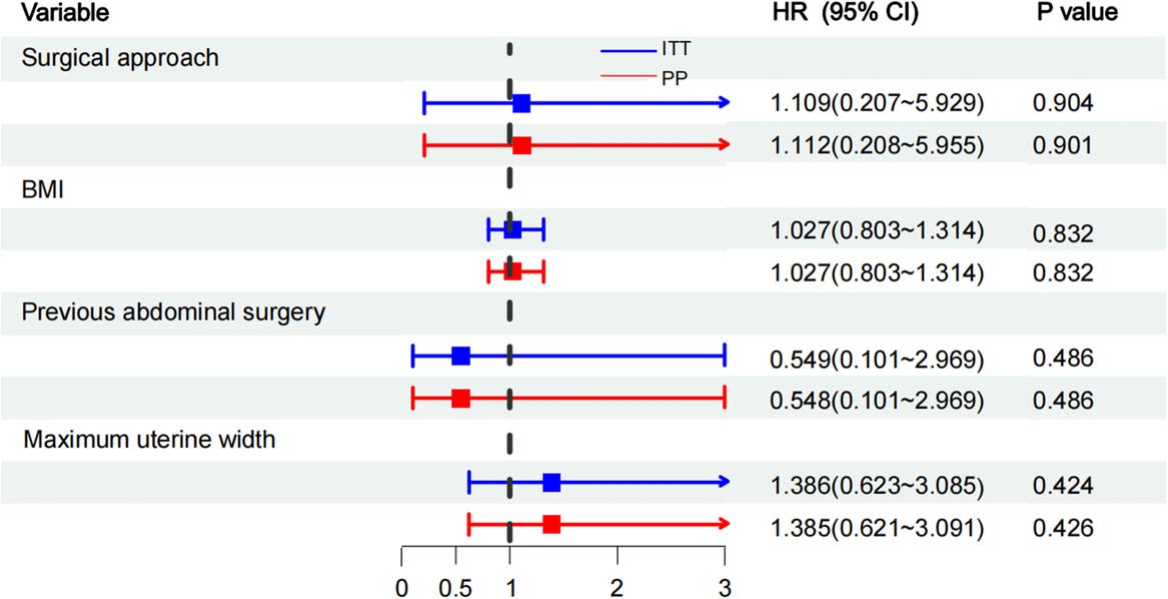
Noninferiority comparison of the detection rate of sentinel lymph nodes between vNOTES and multiport laparoscopy.

**Figure 3:**
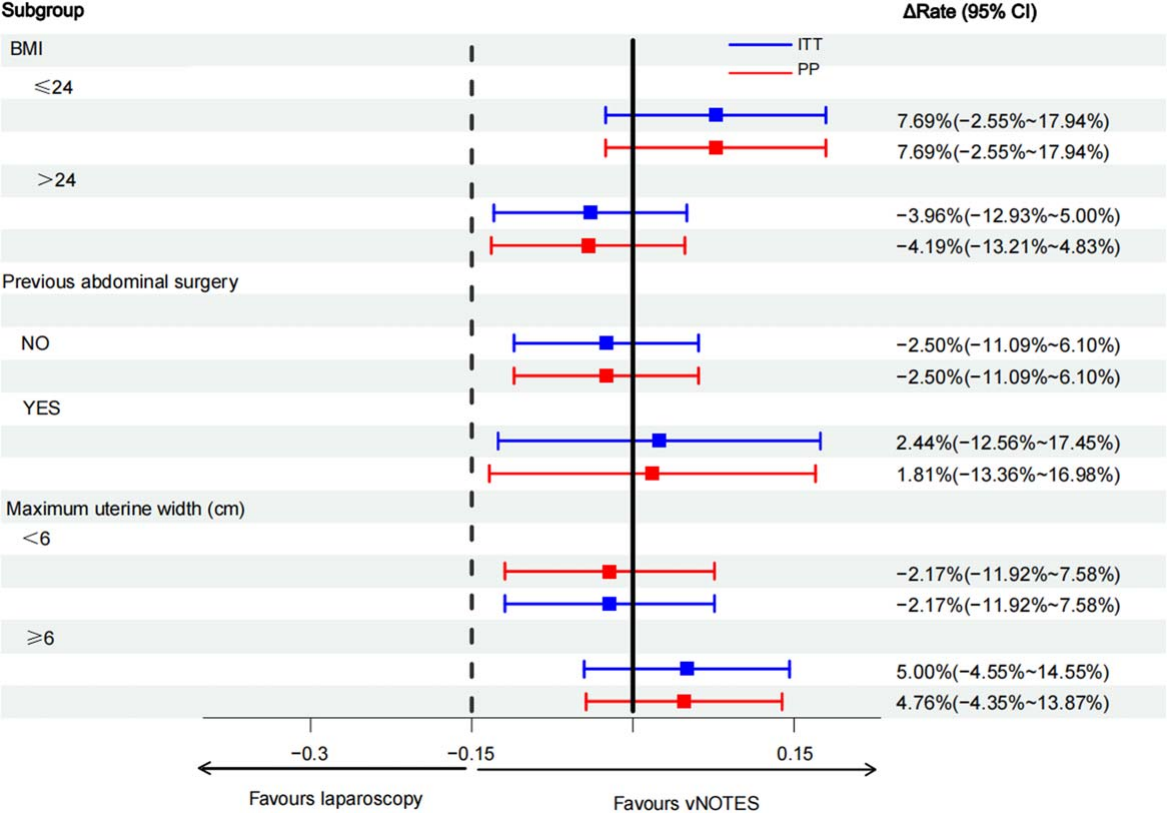
Multivariate logistic regression analysis of risk factors related to the detection rate of sentinel lymph nodes.

The detection rate of a pelvic lymph node on at least one side (PSDR) was 94.73% in the vNOTES group and 96.82% in the laparoscopic group. The bilateral detection rates in the vNOTES and laparoscopic groups were 82.46 and 84.13%, respectively. The side-specific mapping detection rates were 88.60 and 90.48% in the two groups. In the vNOTES group, 17.54% of patients with failed detection of SLNs on at least one side underwent re-detection with laparoscopy and still had no developed SLNs found. The PSDR, SSDR, and BDR of vNOTES were not worse than those of multiport laparoscopy at a noninferiority cutoff of –15%. The lower bound for a rate difference greater than –15% of the 95% CI satisfied the noninferiority assumption (Fig. 2). In the logistic regression model using the BMI, history of abdominal surgery, and maximum uterine width as covariates, the surgical approach was not an independent influence on the detection rate of SLN (Fig. 3). Sensitivity analysis showed that this result remained consistent between the intention-to-treat and per-protocol populations. In the subgroup analysis, the PSDR with vNOTES was no worse than that with multiport laparoscopy (at a noninferiority cutoff of –15%), its result was consistent with the results of the intention-to-treat and per-protocol groups for patients with different BMIs, uterine widths, and histories of abdominal surgery (Fig. 1).

The distribution of total SLNs (*n*=518) by anatomic location in the two groups was as follows: external iliac (*n*=186, 35.91%), obturator (*n*=297, 57.34%), internal iliac (*n*=18, 3.5%), common iliac (*n*=15, 2.9%), and presacral (*n*=2, 0.39%). There was no significant difference in lymph node distribution between the two groups (*P*=0.318). Only one micrometastasis was found in an SLN harvested by vNOTES in the left obturator area.

The perioperative outcomes are presented in Table 2. The median operation time, calculated from the beginning of the intraperitoneal procedures to skin closure, was 132.35 min in the vNOTES group and 138.73 min in the laparoscopic group (*P*=0.362). The median estimated blood loss was 75 ml in the vNOTES group and 50 ml in the laparoscopic group (*P*=0.096). No intraoperative or postoperative complications of Clavien–Dindo grade III or higher occurred in either group. The median postoperative pain scores on the numerical rating scale were 2 and 3 at 12 h and 1 and 2 at 24 h after operation in the vNOTES and laparoscopic groups, respectively, showing that the pain in the vNOTES group was significantly less than that in the laparoscopic group. The median time to first flatus was 17.5 h for the vNOTES group and 21.0 h for the laparoscopic group. In addition, the median postoperative hospital stay was 5 days in the vNOTES group and 6 days in the laparoscopic group, and a significant shortening was observed in the vNOTES group. The medical costs showed no significant difference between the two groups (*P*=0.879).

**Table 2:** Comparison of perioperative outcomes for all patients according to surgical approach.

Variable	vNOTES (*n*=57)	Multiport laparoscopy (*n*=63)	*P*
Operation time, min	132.35±37.91	138.73±38.38	0.362
Estimated blood loss, ml	75 (50–100)	50 (50–100)	0.096
Complications (C-D III and above)	0	0	
NRS pain scores			
12 h	2 (2–2.5)	3 (2–4)	<0.001
24 h	1 (1–1)	2 (1–2)	<0.001
Time to first flatus, hours	17.5 (13.0–20.5)	21 (17.0–33.0)	<0.001
Postoperative hospital stay, day	5 (3–6)	6 (5–8)	0.001
Costs, $	3539.47 (3280.68–6268.47)	3576.89 (3282.06–4432.99)	0.879

## Discussion

This multicenter prospective study, which is based on our previous retrospective study on vNOTES staging for endometrial carcinoma, further confirms the validity and safety of the vNOTES procedure in selected malignant diseases. Consistent with the results of our previous study, we found that the SLN detection rate of patients with early-stage endometrial cancer in the vNOTES group was not different from that of patients in the conventional laparoscopy group. The vNOTES group had advantages such as lower pain scores, shorter time to first flatus, and shorter hospital stays.

SLN biopsy can minimize surgical damage to the lymphatic system without compromising staging and avoid complications such as secondary lymphedema, lymphoid cysts, and lymphatic leakage^15,16^. Given the significance of SLN biopsy in staging endometrial carcinoma, the detection rate of SLNs is an important sign to judge whether a staging surgery procedure is successful^17,18^. In our previous retrospective study, the detection rate of SLNs in the vNOTES group was 87%, and the main reason for failed detection was the inability to visualize SLNs^12^. Some studies suggested that SLN detectability could have been influenced by the various surgical approaches. To further confirm whether failed SLN detection is affected by the vNOTES visual angle or nonidentified SLNs, we converted the cases to conventional laparoscopic surgery for further observation in this prospective study. The results showed that laparoscopy did not identify more SLNs than vNOTES, confirming that the surgical pathway did not affect the detection rate of SLNs.

Although vNOTES technology has undergone substantial development, it remains limited in some difficult operations; still, unlike traditional laparoscopy, it can be used to perform almost all gynecological operations^19^. Therefore, the selection of proper cases and consideration of the patient’s benefit should be focused on in further studies. As we mentioned before, lymph node dissection by vNOTES in our previous studies did not confer real benefits to patients, primarily due to its shortcomings of a long operation time and increased bleeding^12^. Therefore, in this prospective study design, we abandoned adherence to vNOTES and adopted traditional laparoscopic lymph node dissection for cases in which SLN was unidentified. This did not affect the primary endpoint of our study. Moreover, some studies have suggested that if there are no high-risk factors according to pathological examination of frozen uterine tissue during the operation and the preoperative imaging evaluation, lymph node assessment can be omitted in cases were SLN are not identified^20,21^. Therefore, in the real world, physicians may have more options as long as the interests of the patient are fully considered.

Some studies indicated that a retroperitoneal vNOTES approach provided better exposure to the pelvic lymph nodes in the deep inguinal region^9^. However, neither the fluorescence imaging for robotic endometrial SLNB (FIRES) study nor the pelvic SLN detection in the high-risk endometrial cancer (SHREC) trial found deep inguinal SLNs^18,22^. In our study, no identified lymph nodes were found in this region, and the result was the same even for observation using laparoscopy. Another advantage mentioned in the retroperitoneal studies was that SLNs were performed as the first surgical step without the need for a previous hysterectomy. Theoretically, a previous hysterectomy might increase the possibility of leakage of fluorescent imaging agents and affect the localization of SLNs. However, leakage may also occur when the lymphatic vessels are cutoff to create a retroperitoneal space, and substantial damage can occur when the lateral bladder space is opened. The relevant studies at present are case reports or small sample size retrospective studies^7,9,23^; therefore, the retroperitoneal approach needs further evaluation.

There are still many other interesting subjects relevant to the staging surgery of endometrial cancer by the vNOTES procedure, such as the feasibility and safety of collecting peritoneal lavage fluid and harvesting the identified paraaortic lymph nodes. Although it is generally agreed that positive peritoneal cytology does not affect the results of staging, it plays a certain role in the prognosis of patients^24^. In the vNOTES group, we collected peritoneal lavage fluid as soon as the peritoneum was opened from the posterior fornix of the vagina, and compared with that collected from the laparoscopic group, the positivity rate was the same. Because the lymphatic drainage of endometrial carcinoma has three paths, whether the paraaortic lymph nodes may be identified as SLNs is a subject of concern for many clinicians^25^. As demonstrated in the FIRES study, there are few cases of early endometrial cancer in which the paraaortic lymph node are the first site of metastasis^8^. The same result was found in our previous two studies; that is, only one case had a paraaortic lymph node identified as an SLN among a total of 89 SLN mappings.

This study had several strengths, including its rigorous prospective design and its ability to assess the validity of SLNB in vNOTES staging for endometrial carcinoma. However, this study also had limitations. First, our study was carried out in southern China, where high BMIs are relatively uncommon, and it is therefore unknown whether the results can be generalized to obese patients; however, the literature suggests that implementation of vNOTES in obese patients would be beneficial^26^. Second, our estimates of the detection rate may not be generalizable to less experienced surgeons and lower-volume centers, and the young generation of newly trained ‘laparoscopy-only’ doctors will also need to master the skills of vaginal surgery. In addition, this study did not involve the oncological outcomes of the patients, which will be the focus of our future research.

## Conclusion

This prospective multicenter study further confirmed the effectiveness and minimally invasive advantages of vNOTES in staging surgeries for endometrial cancer. Our findings were consistent with the conclusions of our previous observational and retrospective studies. These preliminary explorations have indicated that early endometrial cancer may be the first malignant tumor possessing for which vNOTES surgery may be applicable. Further studies with larger sample sizes are required to evaluate the long-term survival outcomes.

## Ethical approval

1. Ethics committees/Institutional Review Boards of the First Affiliated Hospital of the Army Medical University (KY2020094).
2. Ethics committees/Institutional Review Boards of Shenzhen Hospital, the Chinese Academy of Medical Sciences, and Peking Union Medical College (KYKT2021-9-2).

## Sources of funding

This study was supported by the Chongqing Health Appropriate Technology Promotion Project (2021jstg010)/Chongqing Technology Innovation and Application Development Special Key Projects CSTB2022TIAD-KPX0154. This study was also supported by the Shenzhen Science and Technology Innovation Commission(JCYJ20210324125408022) and the Major project of the National Cancer Center/National Clinical Research Center for Cancer/Cancer Hospital & Shenzhen Hospital, the Chinese Academy of Medical Sciences and Peking Union Medical College (SZ2020ZD007).

## Author contribution

Y.W. and L.S.: concept and design; L.D.: drafting the manuscript; Y.D.: statistical analysis; Y.W. and L.S.: obtained funding. Acquisition, analysis, or interpretation of data, critical revision of the manuscript for important intellectual content, administrative, technical, or material support is done by all authors.

## Conflicts of interest disclosure

The authors declare that they have no financial conflict of interest with regard to the content of this report.

## Research registration unique identification number (UIN)

Clinical Trials.gov Identifier: ChiCTR2000040546.

## Guarantor

Yanzhou Wang and Li Sun.

## Data availability statement

Due to the sensitive nature of the questions asked in this study, survey respondents were assured raw data would remain confidential and would not be shared.

## Provenance and peer review

Not commissioned, externally peer-reviewed.
